# Commonplace

**Published:** 1890-09-27

**Authors:** 


### The Doctor's Pulpit.

### COMMONPLACE.

A religious newspaper, speaking of the late Canon Liddon as a preacher, makes the assertion that he was in the habit of dealing largely in what was commonplace. The writer of the article, himself presumably a preacher, goes on to say that whilst the public, even the educated public, admire and prefer commonplace when it is earnestly handled, preachers, when they listen to another preacher, look rather for "freshness," and think comparatively little of the orator if he is not "fresh." If this preacher's dictum may be accepted as authoritative for the rest of his fellows, we have a very curious standard of value set up in matters religious. Novelty, not truth, is what the preacher likes in religious teaching. A man may be true or not: that is of secondary importance—but he must be new.

Now if this standard be good for religion, it ought also to be good for physic, and for general science, and for every-day business. But in physic it is seen at once to be ridiculous. There are two or two dozen ways, it may be, of treating an ordinary case of typhoid fever. One of the two has been used by the doctor numbers of times with success. The other has never been used by him at all, and has nothing to recommend it except its novelty. According to our preacher the mere fact of its newness should cause the medical man to decide in favour of the untried method. For our part, the standing rule would be always to use first a method which had proved successful before, and if that failed, then to try something new. And our reason would be that the safety of the patient is the first consideration, not the display of the doctor's learning. By this rule, the preacher who sets up freshness as the first object to be aimed at in preaching is either an unintelligent blockhead or a worthless fellow. It is easy to understand why Canon Liddon dealt largely in the commonplace. His object was neither to astonish nor to please his hearers, but to convince their minds and to rouse them to action. That being so, he used language, and illustrations, and familiar knowledge, in order that men and women might easily follow his arguments, and willingly accept his conclusions. Had he devoted himself to the production of freshness for the sake of freshness, he would have been on a level with the mountebank and the play-actor. As it was, he rose to the elevation of the inspired prophet and the consecrated priest.

The preacher who thus puts in his plea for freshness has a great many companions in these times. How it may be among lawyers we do not profess to know, but with doctors novelty is a perfect mania. "But, surely," the reader may protest, "there can be no reason why new methods of treatment should not sometimes be tried in practice? Your present methods are not so absolutely perfect and certain that nothing new is ever to be thought of." Assuredly not. On the contrary, many of our methods are of very feeble efficiency, and some of them fail entirely to accomplish any good result. But our point is that the thing to be always and everywhere aimed at is the cure of the patient, not the novelty of the practice. Are there any old methods in use among doctors which are of any value at all? There are a great many such methods. They are quite commonplace and homely; often as well known to the patient as to the doctor. Those methods, we contend, are to be learnt so thoroughly by every medical man that he shall have them at his fingers' ends. He ought to be so familiar with them, and with the results which they produce, that he will have a constant standard of comparison whereby to test the success or failure of new lines of treatment. The general rule in all practice, we repeat, should be that severe cases are to be treated first by some approved method. If the approved method succeeds, there is no reason to try anything different; if it fails, other methods may then be employed.

A man must have a common and ordinary way of carrying on his business whatever that business may be. He must have a common and ordinary way in order that he may do his work easily, skilfully, and as an expert. If he never does the same thing twice over in the same way he never can really know the value of any one particular way as compared with any other. Efficiency, and always greater efficiency; results, and always better results, are the things to be sought for. Only by learning one or two ways thoroughly so as to be able to compare them throughout with other ways can any man reach high efficiency and successful results. Novelty is a very pleasant thing in itself, and freshness has a certain exciting effect. But novelty when set over against efficiency is the mere straw and husk of a thing as compared with the corn and the kernel. Moreover, there are two kinds of novelty, one of which is gold and the other mere bronze. There is the novelty of a perfectly sincere and original character pursuing great ends for their own sakes, and having no regard to the means except in so far as they may conserve the ends; and there is the novelty of the man who plays new tricks on the tight rope, or swims the Channel, or shoots Niagara in a barrel, or fasts forty-two days when some previous showman has only been able to fast forty-one. This latter novelty is the kind of freshness which the critic preacher failed to find in Canon Liddon, and which he will fail to find in all preachers of the very highest class; and not only in all preachers, but in all men of every kind who have reached a high level of conduct.

If it be a man's business in life to amuse, let him amuse, and let him do it with all his force. A theatre of varieties may play a part in busy life which some people think not disserviceable. But it is not the chief duty of the clergyman or the doctor either to amuse or to startle. If there is a serious and definite business in the world it is the doctor's, and the parson's is not second to it. It should never be an object either of the doctor or the clergyman to present novelties. The cure of the body is the one aim of the doctor, and the cure of the soul is the one aim of the clergyman. Every honest art may be used in the furtherance of either of these ends, but not for the sake of the art, only for the sake of the end. Many men have not the strength and intelligence to go straight for the object in view; but the truly capable man always has both. The ornamental and novel preacher is always second-rate, or even lower; and he is essentially commonplace in himself if not in his methods. The truly great preacher may, and generally must, use language, and illustrations, and knowledge which are altogether commonplace. As to his methods, they may be commonplace to the last degree; as to himself, he is in every instance a new human creature, fresh and stimulating as the harvest moon in a clear August night. The capable doctor is in exactly the same case. Every conceivable thing, whether it is new or old, orthodox or heterodox, he will use for the good of his patient. Whether his methods be as old as Hippocrates or as new as Koch, he will not care one atom. He himself, using them with all the clearness of his intelligence and all the ardour of an honest desire to cure, is as far removed from the commonplace as the East is from the West. Of all pitiable creatures, both in the pulpit and the sick-room, save us from the poor moth who is always flickering about the flames of novelty.

